# NKG2D/NKG2-Ligand Pathway Offers New Opportunities in Cancer Treatment

**DOI:** 10.3389/fimmu.2019.00661

**Published:** 2019-03-29

**Authors:** Alexandra Frazao, Louise Rethacker, Meriem Messaoudene, Marie-Françoise Avril, Antoine Toubert, Nicolas Dulphy, Anne Caignard

**Affiliations:** ^1^INSERMU1160, Institut Universitaire d'Hématologie, Hôpital Saint-Louis, Paris, France; ^2^U1015 INSERM-CIC, Institut Gustave Roussy, Villejuif, France; ^3^Assistance Publique–Hôpitaux de Paris, Department of Dermatology, Hospital Cochin, University Paris Descartes, Paris, France; ^4^Univ Paris Diderot, Sorbonne Paris Cité, Institut Universitaire d'Hématologie, Paris, France; ^5^Assistance Publique–Hôpitaux de Paris, Hôpital Saint-Louis, Department of Immunology and Histocompatibility, Paris, France

**Keywords:** NKG2D, natural killer cells, NKG2D ligands, tumor immunosurveillance, NKG2D CARs

## Abstract

The antitumor functions of NK cells are regulated by the integration of positive and negative signals triggered by numerous membrane receptors present on the NK cells themselves. Among the main activating receptors, NKG2D binds several stress-induced molecules on tumor targets. Engagement of NKG2D by its ligands (NKG2D-Ls) induces NK cell activation leading to production of cytokines and target cell lysis. These effects have therapeutic potential as NKG2D-Ls are widely expressed by solid tumors, whereas their expression in healthy cells is limited. Here, we describe the genetic and environmental factors regulating the NKG2D/NKG2D-L pathway in tumors. NKG2D-L expression is linked to cellular stress and cell proliferation, and has been associated with oncogenic mutations. Tumors have been found to alter their to NKG2D-L expression as they progress, which interferes with the antitumor function of the pathway. Nevertheless, this pathway could be advantageously exploited for cancer therapy. Various cancer treatments, including chemotherapy and targeted therapies, indirectly interfere with the cellular and soluble forms of NKG2D-Ls. In addition, NKG2D introduced into chimeric antigen receptors in T- and NK cells is a promising tumor immunotherapy approach.

## NKG2D/NKG2D-Ls in Tumor Immunosurveillance

The immunosurveillance theory described by Robert Schreiber in 2002 (1) suggested that NK cells are involved in the early control of tumor development, before the successive equilibrium and escape phases when tumor-induced immunosuppression results in the emergence of immune-resistant tumor variants. During the elimination phase, NK cells detect and kill emerging transformed cells. NK cells naturally express receptors detecting stress-induced molecules and altered expression of Major Histocompatibility Complex (MHC) class-I molecules on transformed targets (2). NK cells also potentiate the adaptive immune response through cytokine secretion and by stimulating dendritic cells (DC), notably within lymph nodes (3–8).

Several groups have used samples obtained after curative resection to investigate the role played by NK cells in primary solid tumors. Their results indicated that NK cell infiltrates may correlate with clinical outcome (9–14). In most reports, NK cells were present at low numbers within tumors, as reviewed in (15). In contrast, in colorectal carcinoma (16), lung cancers (14), and gastrointestinal stromal tumors (GIST) (17) numerous NK cells were present in peritumoral areas. NK cells are generally underrepresented among tumor-infiltrating lymphocytes compared to their circulating proportions, and their effector functions are also altered within the tumor microenvironment, as shown in breast and lung malignancies (18, 19). NK cell dysfunction, based on reduced cytotoxicity and cytokine release, correlates with downregulation of NK-activating receptors (18–21). However, this is a tumor-specific phenomenon, described in renal cell carcinoma (RCC) (22), GIST (17), neuroblastoma (23), melanoma (24, 25), and acute myeloid leukemia (26, 27). Altogether, these data revealed that, more than NK cell numbers, expression of NK cell receptors (including activating NK receptors or inhibitory KIRs) strongly influence prognosis and disease outcome. The only notable exception to this conclusion is chronic myeloid leukemia (CML) patients for whom treatment with imatinib was interrupted; in these patients, NK cell numbers were a significant predictive parameter for relapse (28, 29).

In addition, NK cells are thought to play a role in the emergence of metastases, as high numbers of circulating or tumor-infiltrating NK cells inversely correlated with metastatic disease (30, 31). The mechanisms deployed to limit cancer dissemination could involve activating NK receptors, including NKG2D or NKp46. In murine models of melanoma and prostate cancer, IFN-γ production by NK cells triggered by NKp46 activation was found to induce expression of the extracellular matrix protein fibronectin 1 in tumor cells, altering tumor architecture and controlling metastatic invasion (32).

NK cell activation is regulated by signals from activating receptors and inhibitory NK receptors, which bind to HLA-class-I molecules. In addition to natural cytotoxicity receptors, or Ig-type family receptors—the ligands for which have not yet been clearly identified—NKG2D (Natural Killer Group 2, member D)—a C-type lectin receptor—is a major activating receptor for NK cells. In humans, the gene encoding NKG2D (*KLRK1*) lies amid a cluster of genes referred to as the “NK-complex” (NKC) that includes several genes expressed by NK cells [*KLRD1* (CD94), *KLRC4* (NKG2F), *KLRC3* (NKG2E), *KLRC2* (NKG2C), and *KLRC1* (NKG2A)]. NKG2D was first identified on the surface of NK cells as an immunosurveillance receptor. It is also expressed by most CD8^+^ and a small subset of CD4^+^ cytotoxic αβ T cells, as well as innate-like immune cells, such as some iNKT cells and γδ T cells (33, 34). NKG2D is a type II transmembrane protein and, in humans, it associates with the transmembrane domain of the adaptor protein DAP-10 (DNAX-Activating Protein 10). Ligand binding causes dimerization of two NKG2D monomers to form an active receptor which phosphorylates DAP10 and triggers NK cell activation signaling pathways which promote Ca^2+^ influx, actin-based cytoskeleton reorganization, and microtubule polarization (35). This signaling cascade leads to the release of the contents of cytolytic granules, and in some cases elicits the production of cytokines by NK cells (36). NKG2D provides co-stimulatory signals in activated T cells (37). NKG2D expression in NK cells and CD8^+^ T cells can be upregulated, in particular in response to cytokines, such as interleukin (IL)-2, and IL-15, while transforming growth factor (TGF)-β can decrease NKG2D expression.

Our current understanding of the role played by NKG2D in controlling tumor development through NK cell and cytotoxic T-lymphocyte (CTL) activity was aided by the early characterization of its ligands (38). NKG2D binds to eight molecules, members of the following two families: MHC class-I-related chains A or B (MICA/B), and UL16-binding proteins (ULBP1-6). Seven *MIC* genes (*MICA* to *MICGI*) have been identified, located on chromosome 6p21.33. Only *MICA* and *MICB* genes are translated into proteins. As to ULBP, six protein-coding genes (*ULBP1* to *ULBP6I)* have been described on chromosome 6q24.2–25.3. Among all these proteins, MICA, MICB, ULBP4, and ULBP5 are transmembrane-anchored glycoproteins, whereas ULBP1, ULBP2, ULBP3, and ULBP6 are bound to the cell surface by a glycophosphatidylinositol (GPI) motif (39). All NKG2D-Ls are composed of one alpha 1 and one alpha 2 extracellular immunoglobulin (Ig)-like domain, which share a strong homology to the corresponding domains in classical HLA-class-I molecules (40). MICA and MICB contain a third, additional extracellular Ig-like domain (alpha 3). NKG2D-Ls are not associated with β2-microglobulin and bind no antigenic peptide.

## Role of NKG2D and NKG2D-L Polymorphisms in Tumor Immunosurveillance

**Figure 1:**
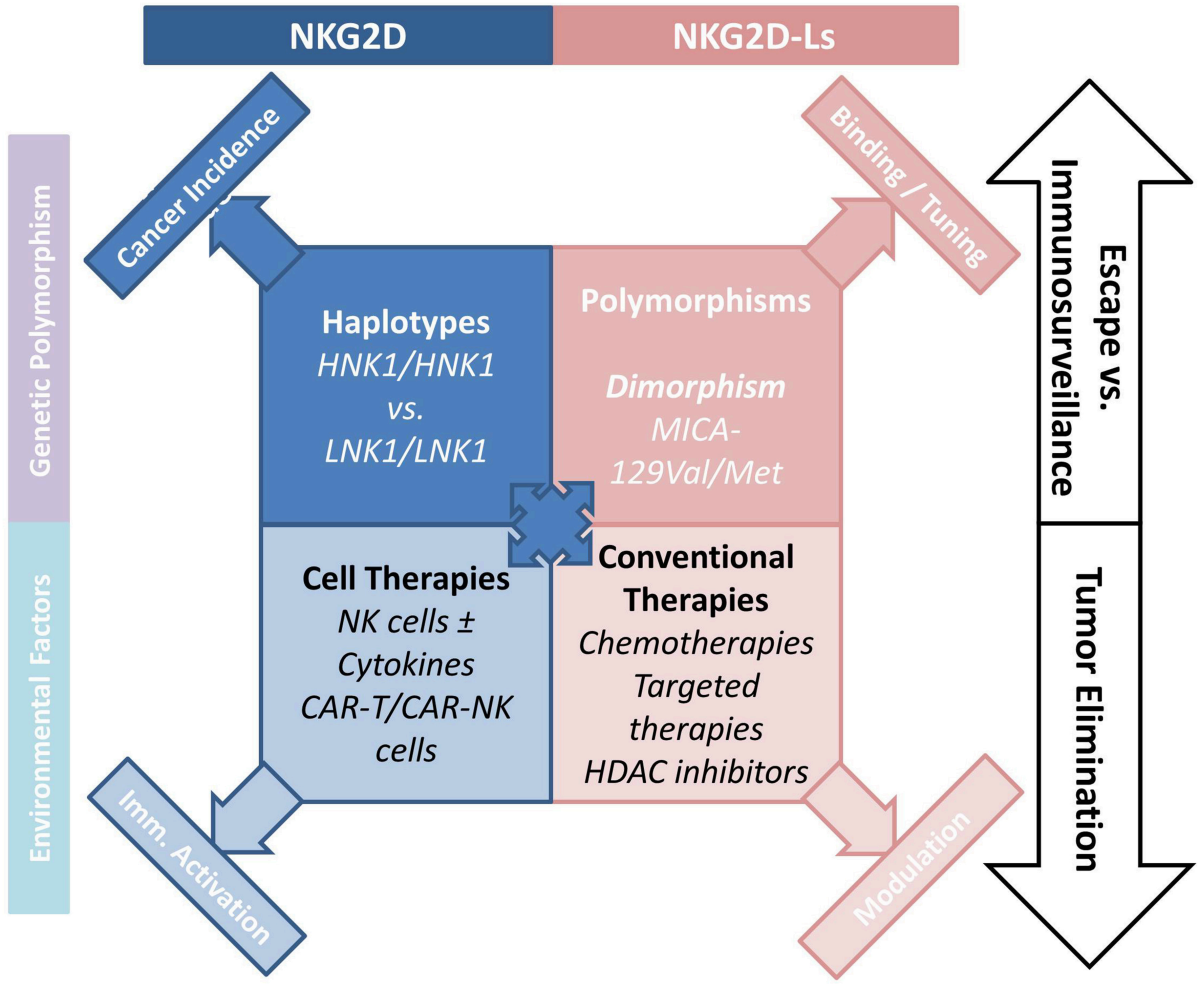
How the NKG2D/NKG2D-L axis is involved NK-cell mediated cancer immunosurveillance. The axis interferes with tumor development and progression, and regulates the antitumor function of NK cells. Various cancer treatments including chemotherapy and targeted therapies indirectly interfere with the cellular and soluble forms of NKG2D-Ls, emphasizing the interest to which this pathway is involved in cancer therapy. Novel CAR-T- and CAR-NK-cell-based therapies hold considerable promise.

The pioneering work by Imai et al. attributed a major role to NKG2D polymorphisms in cancer immunosurveillance (41), and in the prevention of cancer formation (42). An 11-years follow-up survey of a cohort including >3,500 members indicated that medium and high natural cytotoxic activity of peripheral-blood lymphocytes was associated with a reduced cancer risk, whereas low natural cytotoxicity correlated with a higher incidence of cancer. These findings suggest a role for NK cell-mediated immunity in controlling cancer. Analysis of 25 SNPs reported with an allele frequency of >10% in the NKC gene cluster identified eight SNPs in the NKG2D locus (*KLRK1*) that form two haplotype blocks (NKG2Dhb1 and hb2). Each of these blocks can generate two major alleles linked to low (LNK) or high (HNK) cytotoxic activity. Patients with the HNK1/HNK1 NK2GDhb1 haplotype had a lower incidence of cancer compared to those with the LNK1/LNK1 haplotype (41). In a Japanese population the HNK1/HNK1 genotype was associated with decreased colorectal (43) and aerodigestive tract cancer (44). A recent report indicated that NKG2D gene polymorphisms also correlated with control of CML by dasatinib (45). Thus, patients with the NKG2D HNK1/HNK1 haplotype achieved deep molecular response (MR4.5) more quickly than those with other haplotypes. Interestingly, phosphorylation of VAV1 on Tyr174, which was proposed as a major mechanism by which dasatinib intensifies NK cell activity (46), could also be enhanced by expression of the NKG2D HNK1 allele (45). These data suggest that the NKG2D HNK1/HNK1 haplotype may influence cancer development and modulate treatment response (**Figure 1**).

Importantly, MICA and MICB as well as ULBP molecules are highly polymorphic and allelic variation can alter their expression levels or their affinity for NKG2D. As a result, NKG2D-L polymorphisms may strongly influence NKG2D-mediated NK cell triggering by tumor cells or other stressed targets (47). To date, 107 MICA and 47 MICB alleles have been described (updated allele numbers can be found at http://hla.alleles.org/nomenclature/index.html). SNPs are located within regions encoding the α1 and α2 extracellular domains. These alleles are transcribed to produce a total of 82 MICA and 30 MICB proteins. The impact of MICA polymorphisms on protein expression and function remains only partly characterized, and MICA-129 is the only SNP described so far that affects NKG2D receptor affinity (48). MICA-129 (rs1051792) dimorphism—the substitution of a methionine (Met) for a valine (Val) at position 129—alters MICA affinity for the NKG2D receptor: MICA-129Met has an affinity 10- to 50-fold higher than MICA-129Val. Significantly, expression levels for MICA-129Met isoforms are reduced compared to the MICA-129Val molecule (49), but they nevertheless have a higher capacity to trigger the NKG2D pathway, leading to enhanced NK cell activity (50).

Several studies implicated MICA polymorphisms in viral infections and autoimmunity [reviewed in (51)], but few have investigated the impact of MICA polymorphism in cancers. Initial studies focused on cervical cancer and found no association between MICA polymorphism and disease susceptibility (52). In contrast, MICA polymorphism was found to be a significant risk factor for other tumors. For example, MICA-129Val is associated with poor prognosis in nasopharyngeal carcinoma (53) or breast cancer (54) in a Tunisian population. In melanoma, Isernhagen et al. (49) showed that MICA-129Val-homozygous melanoma cell lines expressed higher surface levels of MICA than cells with the Met/Met genotype, which released more soluble MICA. A group of frequent MICA alleles, named MICA-A5.1 (prototype MICA^*^008), produce a truncated protein that acquires a GPI anchor allowing it to be recruited to exosomes, from where it can downmodulate NKG2D expression (55). A GWAS study in cervical cancer patients linked one MICA-adjacent region to the disease and identified a SNP (rs2516448) linked to the MICA-A5.1 frame-shift mutation, suggesting that this allele may cause impaired immune activation resulting in cancer development (56). In colorectal liver metastases, in contrast, the MICA-A5.1 polymorphism was associated with better tumor control and response to treatment (57).

Few studies have investigated ULBP polymorphisms, probably because of the limited number of SNP identified within these genes. However, one ULBP6 dimorphism (two SNP at positions 106 and 147) plays a significant role in determining affinity of the protein for the NKG2D receptor. The ULBP0602 molecule (which contains Leu^106^ and Thr^147^ in contrast to ULPB0601's Arg^106^ and Ile^147^) binds to NKG2D with a 10- to 1,000-fold higher affinity than other ULBPs. This difference in binding could result in decreased interaction of NKG2D with other ligands; it could thus have a negative effect on NK cell function (58).

## Regulation of NKG2D and NKG2D-L Expression

NKG2D-Ls are rarely expressed by healthy cells, but are induced at the cell surface when the cell is stressed as a result of viral infection or malignant transformation; they are therefore called “induced-self” ligands. NKG2D-L-positive cells are detected and eliminated, mostly by NK cells. Expression of NKG2D-Ls is regulated by several mechanisms, which may be transcriptional, translational or post-translational. Expression of NKG2D-Ls is induced by DNA damage, a characteristic of tumor transformation, which leads to the activation of the ATM-ATR DNA repair pathways (59). In mouse models, hyper-proliferation can also induce NKG2D-L expression through activation of the E2F transcription factor (60). Indeed, following HER2/HER3 or BCR-ABL activation, proliferative signals can induce MICA/B and ULBP expression (61). Cellular stress, such as heat shock, has also been reported to induce heat shock factor 1-mediated MICA/B expression (62, 63).

The NKG2D/NKG2D-L pathway is triggered early in cancer development and participates in the elimination of tumor cells. However, during tumor progression, profound changes occur and the NKG2D and/or NKG2D-Ls are targeted by a range of tumor escape mechanisms. Cancer can sculpt the immune environment by selecting immune-ligand-negative variants (1, 64). Tumor cells expressing high levels of NKG2D-Ls can thus be eliminated as part of the tumor immunoediting process, which involves NK cells and NKG2D, and progressively results in the emergence of NKG2D-resistant variants (65). Persistent NKG2D-L expression by tumor cells may cause systemic immunosuppression as a result of NK exhaustion and perturbation of the immune synapse (26). Epigenetic and transcriptional regulation mechanisms are often perturbed in tumor cells, and NKG2D-L expression may be altered. In melanoma, endoplasmic reticulum stress can reduce MICA transcription by modulating activation of the transcription factor E2F1 (66). Indeed, immature isoforms of MICA are retained in the endoplasmic reticulum, resulting in limited membrane-display of MICA (67). Tumors can also inhibit NKG2D-L expression by altering cell surface glycosylation (68), notably in a hypoxic environment (69). In CML, BCR/ABL controls MICA expression through post-transcriptional mechanisms (70), including MICA glycosylation (71). Finally, histone deacetylases (HDAC) may also regulate NKG2D-L expression (72, 73).

Soluble factors secreted by tumor cells and cells from their microenvironment can also alter NKG2D-L expression levels. Thus, TGF-β and IL-10 secreted by regulatory T cells (Tregs) and myeloid-derived suppressor cells downmodulate the expression of NKG2D-Ls (74, 75). Some tumors were demonstrated to secrete TGF-β, resulting in reduced NK cell-mediated lysis (10). ADAM metallopeptidases can catalyze shedding of NKG2D-Ls from the cell's surface, and the released soluble forms can hamper NKG2D signaling (76). Upregulation of ADAM10 or ADAM17 expression in tumors has been linked to the release of solMICA/B, decreased membrane MICA/B expression, and reduced NKG2D expression on NK cells or CD8 T cells (77, 78). Importantly, high serum levels of soluble MIC associated with poor clinical prognosis and the emergence of metastases in RCC (79) and prostate cancer (80). Most leukemia patients present high levels of at least one solNKG2D-L, associated with reduced NKG2D expression by NK cells and impaired anti-leukemic function. In patients who entered complete remission following treatment, solNKG2D-Ls were no longer detected, and NK function was restored (81). Following publication of these data, a meta-analysis of 19 studies, comprising 2,588 patients with 10 different types of tumor, showed that serum concentrations of solMICA/B represent a potential prognostic marker in human cancer (82). In metastatic melanoma, levels of solULBPs are associated with reduced survival in patients treated with immune checkpoint blockers (83); solNKG2D-Ls could thus be a relevant biomarker to select melanoma patients for immunotherapy.

## Conventional Cancer Treatment and the NKG2D/NKG2D-L Pathway

The frequent and high expression of NKG2D-Ls by tumor cells in various human cancers, and the potent antitumor function of the NKG2D/NKG2D-L pathway are now established (Figure 1). Cancer therapies aiming to improve or restore NK- and T-cell responses through NKG2D activation have attracted considerable interest. Among the options proposed, conventional cancer treatments can be used to increase NKGD2 expression and signaling. Indeed, a number of cancer treatments increase NKG2D-L expression on tumor cells. Thus, HDAC inhibitors (Valporic acid) acting on epigenetic regulation of NKG2D-Ls can upregulate their membrane expression (84, 85). Cisplatin, Gemcitabine, Oxiplatin, or 5-fluorouracil chemotherapies have all been shown to increase MICA/B expression on tumor cells through modulation of the ATM-ATR pathway. Other chemotherapies directly kill tumor cells while also inducing NKG2D-L expression, which stimulates tumor elimination by activating immune processes (86–89). Proteasome inhibitors inducing ULBP2 expression and decreasing HLA-I molecules may promote NK cell immunosurveillance of hematologic malignancies (90). ADAM10 or MMP inhibitors could be used to inhibit NKG2D-L-shedding and promote NK cell activity, this approach has been shown to restore NKG2D-Ls in some cancers (91–93). Similarly, antibodies targeting the proteolytic site can prevent shedding of MICA/B proteins, and was shown to limit tumor growth and reduce the formation of metastases in a humanized murine melanoma model (94).

Inhibitors of oncogene-driven mutations are actively being developed to treat various tumor types. Such inhibitors decrease the constitutive activation of kinases involved in tumor cell proliferation and also affect NKG2D-L expression (61, 95). Treatment with imatinib controls the expression of NKG2D-Ls and membrane ganglioside (GM1), and was shown to interfere with NK cell recognition and cytolysis of BCR/ABL cells (70, 71). Treatment of melanoma cells with BRAF and MEK inhibitors modulates the expression of MICA/B and ULBP2, attenuating their recognition by NK cells (96). These effects can be overcome by the simultaneous application of HDAC inhibitors, restoring NKG2D-L expression and stimulating NK cell recognition and function (97). Erk activation was shown to increase NKG2D-L expression (98, 99), but, by acting on MMP, Erk/MEK activation can disrupt the equilibrium between membrane and soluble isoforms of NKG2D-Ls and alter NKG2D function (100, 101). It would therefore be relevant to control membrane and solNKG2D-Ls when performing immunotherapy trials.

## Exploiting the NKG2D/NKG2D-L Pathway for Cell Therapy-Based Cancer Treatment

One of the most promising approaches to cancer immunotherapy relies on revisited immune-cell-based therapies, T cells engineered to express chimeric antigen receptors (CARs) can be highly tumor-specific and have a high killing potential (102). CAR-T cells present a major clinical benefit for patients with malignant hemopathies. For instance, CAR-T cells were shown to be potent against CD19-expressing hematologic tumors in several trials (103, 104). However, the safety of CAR-T cells remains a major obstacle, and CARs must be optimized to increase efficacy and limit treatment-related morbidities due to cytokine-release syndrome (105). Up to now, the use of CAR-T cells for treating solid tumors has been limited by the lack of appropriate tumor antigens (106). Production of modified NK cells expressing CARs could represent an alternative for treatment of solid tumors as they recognize numerous tumor cell types. In addition, infusion of large numbers of NK cells is known not to induce autoreactivity. CAR-NK cells could thus be used in complement or as an alternative to CAR-T cells (Figure 1). NK cells have recently been engineered with CARs to enhance their killing activity, and trials of these cells for treatment of refractory solid tumors have been initiated (107). NK-92 cells were engineered with tumor antigen-specific CARs (EGFR, EpCAM) and successfully used in xenograft models (108, 109). NK cells have a limited lifespan and do not produce memory cells, as a result the excessive activation observed with CAR-T cells should be avoided. In addition, engineered CAR-NK cell lines could be mass-produced, circumventing the need to generate autologous products for each patient, a process that remains challenging and expensive (110). Another promising option would be to express CARs in human iPSC-derived NK cells. This approach might present several advantages, as it could provide a universal cell therapy product (111).

When considering CAR constructs the NKG2D receptor is worthy of attention. Indeed, as we have seen, NKG2D-Ls are widely expressed by a number of solid human tumors, and their relatively selective expression by transformed cells compared to healthy cells makes them an attractive receptor for CARs constructs in T cells. The extracellular domain of NKG2D is used in different CARs constructs with a view to promoting tumor-reactive T reactions (112–114). However, investigations must be performed with care as NKG2D-ligands can also be expressed by healthy cells (115), potentially leading to significant toxicity (116).

The expression of NKG2D-Ls by myeloid cells, Tregs and endothelial cells in the tumor microenvironment suggests that CAR NKG2D cells could also be used to control *in situ* immunosuppression (117). Thus, CAR-NK cells transduced with NKG2D fused to the TCR CD3z chain could be used to target suppressive myeloid cells and improve infiltration and function of subsequent infusions of tumor-specific CAR-T cells (118).

NKG2D-based CARs with full-length NKG2D or NKG2D-ligand binding domains represent a novel strategy to target several types of solid tumors, and would have the capacity to induce potent antitumor immunity in patients. NKG2D CARs could not only target tumors but also myeloid immunosuppressive cells and Tregs, as well as others cells in the microenvironment that promote tumor progression.

## Author Contributions

AF, ND, and AC wrote the manuscript. AT, M-FA, MM, and LR read and corrected the manuscript.

### Conflict of Interest Statement

The authors declare that the research was conducted in the absence of any commercial or financial relationships that could be construed as a potential conflict of interest.
